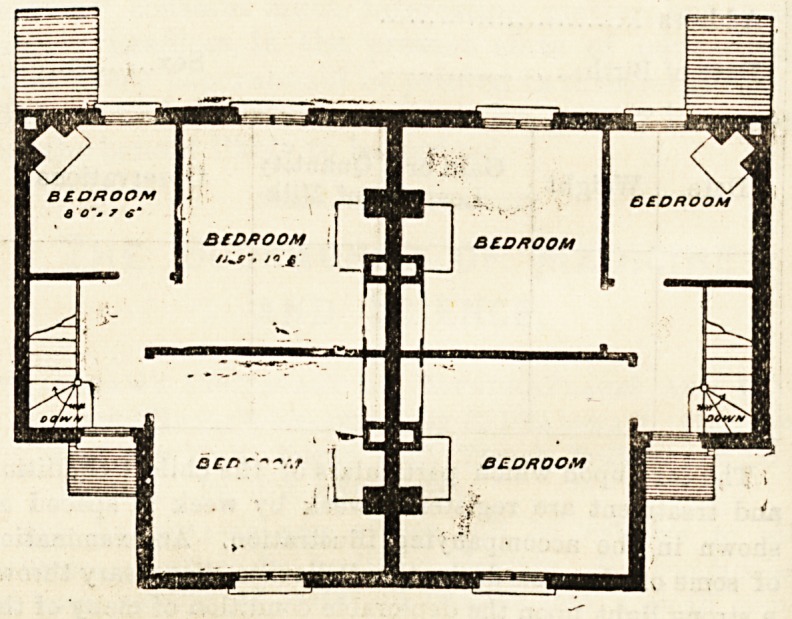# Regimental Homes

**Published:** 1904-10-01

**Authors:** 


### REGIMENTAL HOMES.

Although these regimental homes are not in strict sense hospitals, we think a description of one of them, together with some general details, can hardly be out of place in our columns. The homes are intended for those soldiers who have been bodily disabled; and moreover the principle is an important one, and the work done by the homes is useful in the extreme.

It is pleasing to note that the movement has met with much help from those not directly connected with the Army.

We hear that medical and legal services have been given gratuitously, and that architects have wholly or partly remitted their fees. Lloyds Patriotic Fund have paid expenses incurred in the removal of disabled soldiers to their new homes; railway companies have conveyed families free of charge, and we may go lower down the social scale and state that a sweep who swept the chimneys declined to accept any payment.

In addition to 18 homes already completed, 25 others are either begun or about to be started, and several other regiments are raising funds for the purpose. It is to be hoped that this good work will go on until every regiment in the British Army has at least two or three pairs of these comfortable homes for its disabled men. It has always been with us a matter of much regret that any man who has fought for his country and been maimed in the fight should have to wander about in more or less aimless fashion, and have to pitch his tent wherever circumstances may seem to throw him, instead of having his abode more or less in touch with his old comrades.

Of course the bulk of the money required to build any of these homes comes from the officers of the respective regiments and from those living near the regiment at its territorial habitation. The general committee is a very strong one, with Lord Roberts as president, Lord Brassey as treasurer, Mrs. Papillon as secretary; the bankers are Lloyds, of 16 St. James's Street; and the office is at 11 Tothill Street, Westminster. Any information concerning the movement can be obtained at the Westminster offices.

The plans we give in this issue are those of the home for the East Kent Regiment—the Buffs or the old 3rd Regiment. The site for one pair of these cottage homes was provided by Mr. J. F. Friend, and money for one cottage was collected by Mrs. Friend. Mr. Brooks, of the Convent, presented the funds for the other. The site is a very salubrious one, on the road from St. Peter's to Margate, and the plan is extremely compact and convenient. The entrance to each cottage is at the side, and it opens into a small lobby, where the staircase is placed. The ground-floor has sitting-room, kitchen and scullery, and the first-floor has three bedrooms. The cottages have a party wall, and are alike. The elevation is good, and the salient point is formed by the union of the two sitting-rooms. This part is gabled, and a stone shield, with the regimental badge engraved on it, has been placed between the two front bedroom windows.

## Figures and Tables

**Figure f1:**
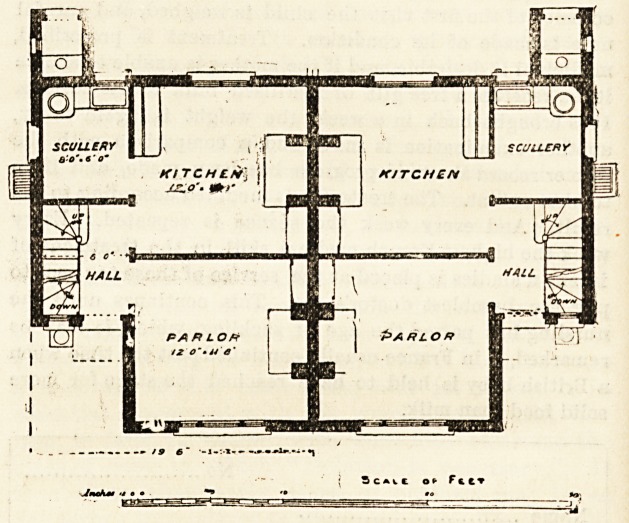


**Figure f2:**